# Mediterranean Diet Combined with Regular Aerobic Exercise and Hemp Protein Supplementation Modulates Plasma Circulating Amino Acids and Improves the Health Status of Overweight Individuals

**DOI:** 10.3390/nu16111594

**Published:** 2024-05-23

**Authors:** Antonio D. Miguel-Albarreal, Fernando Rivero-Pino, Elvira Marquez-Paradas, Elena Grao-Cruces, Teresa Gonzalez-de la Rosa, Sergio Montserrat-de la Paz

**Affiliations:** 1 Department of Medical Biochemistry, Molecular Biology, and Immunology, School of Medicine, University of Seville, Av. Sanchez Pizjuan s/n, 41009 Seville, Spain; antoniodemiguelalbarreal@gmail.com (A.D.M.-A.); emarquez5@us.es (E.M.-P.); egrao@us.es (E.G.-C.); mgonzalez18@us.es (T.G.-d.l.R.); 2 Instituto de Biomedicina de Sevilla (IBiS), Hospital Universitario Virgen del Rocío, CSIC, University of Seville, 41013 Seville, Spain

**Keywords:** amino acids, plant protein, pre-diabetes, obesity, Mediterranean diet

## Abstract

Plant protein is considered a sustainable health-promoting strategy to prevent metabolic syndrome. Lifestyle changes (including dietary patterns and exercise) have been demonstrated to exert an effect on human health by modulating the biochemical status in humans. The objective of this study was to assess whether supplementation with hemp protein within a Mediterranean diet context together with exercise could help to ameliorate the metabolic statuses of patients prone to developing metabolic syndrome. For this study, 23 patients followed with Mediterranean diet and engaged in aerobic exercise according to the WHO’s recommendations, while also being supplemented with hemp protein, for 12 weeks. A comparison of anthropometric, biochemical, and mineral data as well as amino acid values was made between the start and the end of the study, with the subjects acting as their own control group. Statistical analyses included a paired *t*-test, Wilcoxon paired test, Pearson correlation coefficient, and Sparse Partial Least Squares Discriminant Analysis to evaluate significant differences and correlations among parameters. There were statistically significant changes in total cholesterol, HDL-C (+52.3%), LDL-C (−54.0%), and TAG levels (−49.8%), but not in glucose plasma levels. Following the intervention, plasma concentrations of some amino acids, including α-aminoadipic acid, phosphoethanolamine, and 1-metylhistidine, increased, whereas those of asparagine and alanine declined. Different correlations between amino acids and the other parameters evaluated were reported and discussed. A Mediterranean diet combined with regular aerobic exercise, together with protein supplementation, can highly improve the metabolic parameters and anthropometric parameters of subjects with obesity and impaired glucose levels, ameliorating their health status and likely delaying the development of metabolic syndrome.

## 1. Introduction

The definition of overweight and obesity is abnormal or excessive deposits of fat that poses a health concern, and it has become an epidemic worldwide. Values over 25 kg/m^2^ in body mass index (BMI) as considered as overweight, and over 30 kg/m^2^ as obese, although others classification exist according to the waist circumference or the waist-to-height ratio. It must be noted that the associated health care costs of ameliorating the health status of overweight patients are substantial. On top of that, there is a correlation between the current rise in obesity incidence and the increase in type 2 diabetes mellitus (T2DM) prevalence, cardiovascular diseases, and cancers, among others [1]. Nearly 90% of T2DM patients are overweight or obese. Obesity increases the risk of prediabetes and T2DM significantly, particularly when it is combined with increased abdominal and intraabdominal fat distribution and triacylglyceride (TAG) content in the intrahepatic and intramuscular tissues. This is due to the fact that obesity causes β-cell dysfunction and insulin resistance [2]. In this sense, pre-diabetes is the state that precedes T2DM, one of the most prevalent diseases worldwide, defined by insulin resistance, which impairs glucose metabolism [3].

The relationship between diet and health has been widely described. In this regard, the intake of specific groups of food might help to reduce inflammation through different pathways, including the glucose metabolism and, consequently, the development of obesity through specific metabolic pathways. For this reason, in the framework of delaying or preventing the occurrence of increases in weight in adults, nutritional guidelines, such as Mediterranean diet guidelines, are being proposed as a strategy to promote health in subjects while being cost-effective at the same time [4,5]. The importance of preventing the onset of obesity stems from the fact that usually, this condition, generally associated with prediabetes, already includes insulin resistance and a dysfunctional β-cell population. The abnormal blood glucose levels may then upregulate inflammatory marker expression and result in the generation of reactive oxygen species (ROS), which ultimately results in vascular dysfunction. Increased ROS and inflammation may also worsen insulin resistance and impair insulin secretion in a reciprocal manner. Understanding the underlying process, particularly how protein and amino acids affect the metabolic status of diabetes, is important for the study of the transition from pre-diabetes to T2DM [6].

On top of that, numerous extensive epidemiological investigations have demonstrated a correlation between blood pressure and BMI in people who are overweight or of a normal weight. The most successful non-pharmacological therapy strategy for obese hypertension patients has been demonstrated to be weight reduction. A little weight reduction can reduce blood pressure in both hypertension and non-hypertensive people. Losing weight impacts blood pressure, reducing its levels, most likely due to an improvement in insulin sensitivity and a decrease in sympathetic nervous system activity [7]. In line with the relevance of dietary protein in the health status of humans, the current suggested dietary consumption of protein has been established at 0.8 g/kg/day for young healthy men, whereas consumption ranging from 1.0 to 1.5 g/kg/day in older adults has been reported to trigger improvements in glycemic control and muscle mass [8,9]. It has been demonstrated that diets with high contents of protein are beneficial in terms of glycemic markers due to satiety, lowering the postprandial glucose response and increasing thermogenesis and the ability to decrease the rate of muscle protein breakdown. Similarly, exercise has been demonstrated to synergistically help in the prevention of the progression of prediabetes by inducing favorable changes in glycemic control independent of lean body mass increases [9]. In this regard, it has been shown how exercise intervention is efficient in ameliorating inflammatory, metabolic, and lipid markers in middle-aged and older adults with diabetes [10,11]. According to Mthembu et al. [12], patients with T2DM may benefit from therapies like calorie restriction and exercise over the course of two to four months in order to increase their insulin sensitivity, in part by targeting the phosphoinositide 3-kinases/protein kinase B pathway. One interesting source of protein currently gaining interest in the research community is hemp protein. It has been demonstrated to be of high quality and environmentally friendly [13]. In fact, some studies have already shown positive effects of hemp protein in reducing postprandial blood glucose [14], and there is a huge body of evidence supporting that hemp protein is a foodstuff with potential health benefits [15]. To the author’s knowledge, no specific studies have been conducted to evaluate the circulating amino acids in humans after hemp protein ingestion for long periods, which is of interest considering the amount of essential amino acids this protein contains.

Additional research is required in order to elucidate whether the intake of specific protein sources together with healthy lifestyle patterns could further promote an anti-inflammatory response that might lead to healthier statuses for overweight patients, in order to promote healthier ageing. In this study, a nutritional intervention (Mediterranean diet and aerobic exercise) supplemented with hemp protein was carried out in overweight patients in order to evaluate the changes in anthropometric data, biochemical parameters, amino acids in plasma, and minerals after twelve weeks, and how different parameters affected the outcome.

## 2. Materials and Methods

### 2.1. Study Design, Participants, and Eligibility Criteria

A nutritional intervention following the Mediterranean diet, combined with aerobic exercise and supplemented with hemp protein, was conducted. Patients (n = 27) were recruited, and interventions were delivered at a community pharmacy located in the south of Spain. During the study, some of the patients dropped out and were not considered in the results. The final number of subjects was n = 23. The inclusion criteria were: age between 18 and 65 years old; waist circumference ≥ 94 cm in men and ≥88 cm in women; and metabolic equivalent < 4 (calculated through the long version of the International Physical Activity Questionnaire). The exclusion criteria were prediabetic patients on pharmacotherapy; subjects suffering from medical issues (e.g., diabetes or another chronic disease) or physical limitation; smokers; and pregnant or lactating women. The study was conducted according to the Good Practice Guidelines and in line with the principles outlined in the Helsinki Declaration of the World Medical Association. This study was approved by the Investigation Ethic Center (CEI) of Virgen Macarena and Virgen del Rocio Hospitals (Study ID 2246-N-20), and written informed consent was obtained from all participants before entering the study. Clinical Trial Registry number is NCT06129578. Before the assessment, all questions were addressed, and the participants signed informed consent forms. The participant flow chart can be seen in Supplementary Material Figure S1.

### 2.2. Dietary Intervention

All participants followed a low-energy diet (1315 kcal/daily) based on the Mediterranean diet for 12 weeks, supplemented with 20 g/day of hemp protein. The specific menu was designed by a trainee nutritionist. The program prescribed five meals a day, with the time customized to fit the lifestyle of each patient. Based on the baseline metabolism, the energy and nutritional parameters of every patient were computed. Patients reported the dietary intake data daily. The intervention included a face-to-face visit every two weeks, individual nutrition counseling, and education sessions. Hemp protein (Myvegan, 54% of protein, 25% of fiber, 12% of fat, and 10% of carbohydrates) was provided free of charge to the participants. The following is a summary of dietary action guidelines to create the menus for the patients: legumes, two or more servings per week; fruits, three servings of fresh fruits per day; vegetables, two or more servings per day (at least one serving raw); nuts, four nuts per day (15 g); olive oil, 40–60 g per day; fish and seafood, four or more servings per week; white meat, 2 or more servings per week; eggs, 2 or more servings per week; grains and potatoes, one or more servings per week; processed meat, commercial bakery goods, and sweets were not allowed; for alcoholic drinks, occasional consumption was allowed, with, no more than one glass of wine a day; and carbonated beverages were not allowed. Regarding culinary techniques, low-fat cooking methods (microwaving, roasting, boiling, grilling, and poaching) were used. Frying was not allowed.

### 2.3. Physical Activity Intervention

The exercise intervention was adapted from the World Health Organization physical activity recommendations, in which adults should engage in at least 150 min of moderate-to-intense aerobic physical activity per week. The exercise was assessed using a standardized questionnaire (SBQ) adapted to local habits. A smart watch (Xiaomi Mi Band 4) was provided free of charge to all participants. Exercise was monitored using Zepp Life, an app where the patients registered all activities.

### 2.4. Clinical Measurement

Weight (±0.1 kg), metabolic age, and body fat percentage were measured using a TANITA Body Composition Analyzer (Model DC 240 MA, Tanita, Barcelona, Spain). Waist circumference was measured exactly midway between the lowest rib and the peak of the iliac crest. Measurements of systolic and diastolic blood pressure (SBP and DBP) were performed with a standard method. BMI was calculated as weight (kg)/height (m)^2^. Alcohol and tobacco consumption were also registered by directly asking the participants.

### 2.5. Biochemical Measurements and Mineral and Amino Acid Content

After an overnight fast (8–10 h), blood samples were collected at baseline (before the intervention, t_0_) and after the 12-week intervention (t_f_). Blood was collected into Vacutainer tubes for serum with separating gel and into K2EDTA tubes for plasma (Becton Dickinson, NJ, USA). The samples were centrifuged (1400× *g*, 10 min at 4 °C), and serum was frozen at −80 °C until analysis. Fasting blood samples were analyzed at the Department of Medical Biochemistry (School of Medicine, University of Seville). Glucose was measured using the glucose/oxidase method (Glucose GOD-PAP; Biolabo, Madrid, Spain). Total cholesterol and TAG were determined using enzymatic methods (CHOD-PAP and GPO-PAP, respectively; Roche Diagnostics, Basel, Switzerland). High-density lipoprotein-cholesterol (HDL-C) was determined after precipitation with phosphotungstic acid. Low-density lipoprotein cholesterol (LDL-C) was calculated with the Firedewald formula. Mineral content was evaluated using UV or colorimetrics commercial kits (Bioscience Medical, Madrid, Spain). The analyses of the amino acid content in plasma samples were carried out at the University of Sevilla (Microanalysis Research Service, CITIUS) using an amino acid analyzer BIOCHROM 30 (Laborservice Onken, Gründau, Germany), following an amino acid analysis method based on ion exchange chromatography with post-column derivatization with ninhydrin.

### 2.6. Data Preprocessing and Exploratory and Statistical Analysis

Prior to statistical analysis, the identified amino acids were pre-processed, removing all amino acids with >30% missing values. No normalization method was applied. Missing values were imputed by half the minimum of each amino acid, and the overall behavior of the samples was analyzed via principal component analysis (PCA) before and after the pre-processing process. The normality of all data sets was analyzed with the Shapiro–Wilk test [16].

To test whether the intervention had led to significant changes, all parameters and variables that fit a normal distribution were subjected to a paired *t*-test, while those that did not fit a normal distribution were subjected to a Wilcoxon paired test.

In the search for possible correlations of interest, the calculation of Pearson’s coefficient was used as a linear correlation value between variables. Correlation heat maps were produced using the ggplot2 package (v3.4.2) from the correlation matrix between amino acids and biochemical parameters and between amino acids and mineral values [17].

Finally, a sample classification model was generated using Sparse Partial Least Squares Discriminant Analysis (sPLS-DA), which finds discriminant relationships between groups in the multivariate amino acid dataset, also employing a feature selection approach to identify the subset of amino acids that is most relevant in group differentiation. The model was generated using the mixOmics package (v6.22.0), previously setting a seed of 5249 [18]. The optimal number of components and variables was obtained by applying the M-fold method as a cross-validation method with a fold value of 5, and the contributions of the variables in each of the components was analyzed. The optimal number of components and variables was obtained by applying the M-fold method as a cross-validation method with a fold value of 5. A total of 38 samples were randomly selected for model training, leaving the remaining 8 for model evaluation, and performance was evaluated by calculating the area under the curve (AUC) or ROC curve.

## 3. Results

### 3.1. Anthropometric Data

As shown in Table 1, the results of the anthropometric characteristic of all the individuals in the study who completed the intervention are reported. Men’s and women’s values can be found separately in the Supplementary Material, Table S1. According to the forms and the supervised monitorization, all subjects followed the exercise guidelines and the dietary patterns as indicated. Statistically significant differences over time were observed in all the parameters except for beats per minute.

### 3.2. Biochemical Parameters

In Table 2, the results of the biochemical profiles of all the individuals in the study who completed the intervention are reported. Men’s and women’s values can be found separately in the Supplementary Material Table S2. Statistically significant differences were found for all the parameters except for glucose. The percentages of change for HDL-C and LDL-C were 52.3% and −54.0%, respectively, while for TAG, the change was −49.8%, and for total cholesterol, the value was reduced by −29.1%.

### 3.3. Mineral Content

The results of the mineral contents of all the individuals in the study who completed the intervention are reported in the Table 3. Men’s and women’s values can be found separately in Supplementary Material Table S3. Statistically significant changes were observed following the intervention in the contents of calcium and phosphorus, with decreases of −14.2% and −56.7%, respectively, while in the case of chloride, the change reported was a statistically significant increase (+8.6%) after the intervention. The change in magnesium was not found to be statistically significant.

### 3.4. Plasma Circulating Amino Acids

The plasma-circulating amino acids were measured in all the patients before and after the intervention. The total number of amino acids evaluated was 41, but after the processing of the data as described in Section 2.6, only 29 amino acids were included in the report (Table 4). The most significant changes found were α-aminoadipic acid (AAAA, +2587.6%), phosphoethanolamine (Pea, +99.2%), and 1-metylhistidine (Mhis_1, +57.5%) for those that increased, and asparagine (−47.7%) and alanine (−29.9%) for those for which the plasma concentration decreased. The AAAA and Mhis_1 changes were not statistically significant, but the changes observed were high because of the pretreatment and analyses applied to the data. Most of the amino acids conformed to a normal distribution and imputed the missing values by half of the minimum for each amino acid. In the exploratory analysis, PCA revealed the difference in behavior between samples, showing that, after pre-processing, component 1 separated consistently at the two intervention times (t_0_ and t_f_). The PCAs performed before and after pre-processing of the data can be found in Supplementary Material Figure S2. Consequently, amino acid levels in the blood behaved differently in subjects before and after the intervention, and significant differences were found in many of them. To further explore the hypotheses, the multivariate analysis technique (sPLS-DA) was used to create a model capable of classifying and discriminating interventions according to their amino acid contents. The model was fitted with the optimal number of components (3 components) and variables, and it was observed that component 1 was the main component responsible for the separation or distinction of groups, explaining 27% of the variability of the data set (Supplementary Material Figure S3). The evaluation of the model showed AUC values of 0.9622 in the first component, making the high sensitivity of the generated model clear. The construction of the model did show findings of interest; observing the loadings, i.e., the weight of each variable in component 1 (responsible for group separation), we found that the amino acids asparagine, histidine, alanine, and phenylalanine were the four most influential (Supplementary Materials), participating notably in the characterization of the profile prior to the intervention or t0.

### 3.5. Correlation of Plasma Circulating Amino Acids and Biochemical and Mineral Parameters

**Figure 1:**
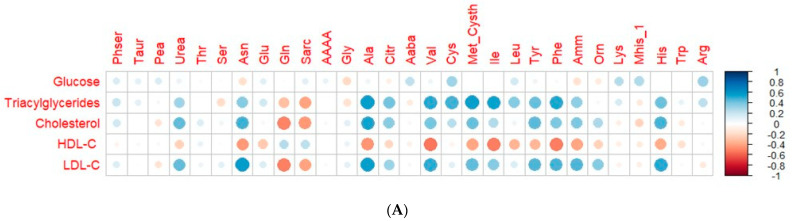

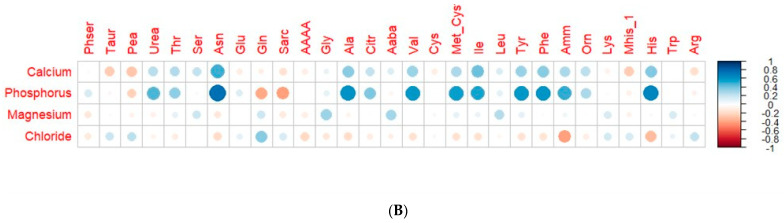
Correlation heat maps. (**A**) Represents the significant correlations (*p* value < 0.05) between some biochemical parameters and the amino acids quantified in the subjects after pre-processing and imputation of values. (**B**) Shows the significant correlations (*p* value < 0.05) between the same amino acids and the mineral levels quantified in the subjects. The color and size of the circle represents the Pearson correlation coefficient. Phser, phosphoserine; Taur, taurine; Pea, phosphoethanolamine; Thr, threonine; Ser, serine; Asn, asparagine; Glu, glutamate; Gln, glutamine; Sarc, sarcosine; AAAA, α-aminoadipic acid; Gly, glycine; Ala, alanine; Citr, citrulline; Aaba, α-aminobutiric acid; Val, valine; Cys, Cysteine; Met_Cysth, methionine+cystathionine; Ile, isoleucine; Leu, leucine; Tyr, tyrosine; Phe, phenylalanine; Amm, ammonia; Orn, ornithine; Lys, lysine; Mhis_1, 1-metylhistidine; His, histidine; Trp, tryptophane.

The connections between the 29 amino acids subjected to statistical analyses and the biochemical parameters and mineral content were investigated as depicted in Figure 1. Strong positive correlations, meaning values higher than 0.5 between several amino acids and all the biochemical parameters, were found, except for glucose and HDL-C. The TAG content was found to have correlations with alanine, valine, methionine+cystathionine, isoleucine, and phenylalanine, being the parameter with more correlations with amino acids. In the case of LDL-C, correlations with asparagine, alanine, valine, and histidine were found at the same that a negative correlation with glutamine was found. On the other hand, the HDL-C content was inversely correlated with valine, isoleucine, and phenylalanine (Figure 1). In the case of minerals, eight detected compounds (asparagine, alanine, valine, methionine+cystathionine, isoleucine, tyrosine, phenylalanine, ammonia, and histidine) were correlated with the content of phosphorus (values >0.5), with asparagine being the one with the highest value (0.76). For the other minerals, no strong correlations, either directly or inversely, were found (Figure 1).

## 4. Discussion

Following a healthy lifestyle (i.e., regular exercise and consumption of healthy foodstuff) is necessary to prevent the development of certain diseases, especially those related to metabolism, such as diabetes or cardiovascular diseases, which are usually associated with the described situation of obesity. Overall, the results presented in this nutritional intervention show its efficacy in regulating the health statuses of overweight patients based on the results of different parameters related to the disease.

Regarding the anthropometric data, the statistical significance observed in the parameters can be directly correlated to both the foodstuff from the dietary intervention and the aerobic exercise [11], as has been demonstrated by several studies demonstrating the health benefits of the Mediterranean diet for the overall health of humans [19,20]. The differences found in some parameters between males and females are likely consequences of the human body’s physiology. Men and women have very different body compositions, as males typically have less fat mass and higher absolute and relative lean muscle mass [21], factors which were considered in the inclusion criteria regarding the weight circumference, for instance.

In general, the individuals exhibited low levels of HDL-C and high values of glucose, cholesterol, TAG, and LDL-C at the start of the intervention (t_0_), which possible indicates their non-healthy conditions [22]. Regarding the biochemical parameters of the target population, the glucose levels did not statistically significantly change over time after the 12 weeks of the intervention (t_f_). This absence of an effect of the diet on glucose levels was also observed in our previous human study, without hemp protein supplementation [23]. Nonetheless, low initial glucose levels do not have to be indicative of good glucose metabolism, but this condition may be associated with hyperinsulinemia. However, considering that insulin levels were not measured in the study, we cannot draw conclusions. On top of that, this absence of variation and relatively low levels of glucose for people with obesity may be indicative of the onset of insulin resistance, which may be reversed with nutritional intervention, because insulin sensitivity is known to improve with diet and weight loss, parameters that were modified in the present study. However, it must be noted that protein supplementation does not necessarily have an effect on blood glucose levels in the long term [24], although some studies have reported that, although it depends much on the source and dosage, eating protein may cause the postprandial state’s glucose levels to decrease [25].

After the intervention, the rest of the biochemical parameters which could be considered biologically relevant were adequately modulated. The decrease in LDL-C was −54.0%, an amelioration of the results after the intervention, which can be associated with both the diet and the aerobic exercise performed by the patients. It has been reported that, especially, the ingestion of pulses, hazelnuts, walnuts, and high-fiber/whole-grain foods can promote a decrease in blood LDL-C levels. On top of being part of the Mediterranean diet, fiber was supplemented with the hemp protein product. However, it must be noted that the changes in LDL-C values were not different in a previous intervention in humans which was similar to the one followed in this study, but without the hemp supplementation [23]. In the case of HDL-C, the increase was also statistically significant after the intervention (+52.3%), most likely also due to the dietary pattern followed [26]. Finally, for the TAG values, the decrease was also statistically significant, contrary to what was found in a similar intervention without hemp protein supplementation [23]. This finding is interesting as it indicated that hemp protein might be exerting extra health benefits compared to the Mediterranean diet plus aerobic exercise only, although validation with more subjects should be carried out. In fact, Rodriguez-Leyva and Pierce [27], who reviewed the cardiac and hemostatic effects of dietary hempseed, concluded that this product might have several health benefits in relation to different diseases, but the effects were discussed to be attributed to the oil fraction. However, it must be noted that the products usually used in nutritional interventions usually contain several nutrients; thus, it is complicated to establish the cause–effect relationship. In addition to the changes in the diet, considering that all the individuals followed the exercise program, an association of this exercise to the improvement of values regarding a healthier status after the 12 weeks can be also made, as exercise has been reported to ameliorate total cholesterol, HDL-C, LDL-C, and TAG in patients with hyperlipidemia [28], indicating synergy with the dietary patterns. However, the levels of contribution of the diet and exercise to the final outcomes cannot be estimated.

Table 3 shows the concentrations of the evaluated oligoelements in the subjects’ serum before and after the intervention. Regarding mineral levels, these are crucial components in maintaining the body’s homeostasis, since many of them serve as enzyme cofactors and because a lack of them may be a risk factor for disease development. According to several studies, the plasmatic concentrations of these might vary depending on the person’s state [29,30]. The contents of the minerals evaluated were found to be significant different in all cases except for magnesium. An increase after the study was observed for chloride (+8.6%), whereas in the case of phosphorus and calcium, the levels were higher at the beginning of the intervention. This decreases in phosphorus (−56.77%) and calcium (−14.2%) meant a reduction in the serum levels up to the range of these minerals’ values. Normal phosphorus values usually range from 3.4 to 4.5 mg/dL and normal calcium values range from 8.5 to 10.5 mg/dL [31]; consequently, the values obtained in the intervention can be linked to an improvement in the health of the subjects, as the values before the intervention were high. In the case of chloride, the statistically significant increase did not imply any biological relevance, as the values were still within the range of what is considered normal [31]. Mariño et al. [32] reported that exercising regularly led to a decrease in magnesium and phosphorus in erythrocytes in young adults compared to subjects not doing the exercise, similar to the decrease in phosphorus reported by Musavian et al. [33] following an active exercise program.

The relevance of the amino acid supplementation relies, on one hand, on the fact the some of them are essential and should be ingested in the diet, and on the other hand, they might exert specific roles in different metabolic pathways, having functions which might still need to be discovered. As an example, it has been reported that amino acid supplementation (essential amino acids plus arginine) for 16 weeks decreases plasma and liver TAG in the elderly [34]; that supplementation with arginine, phenylalanine, and alanine in combination with physical activity facilitates abdominal fat reduction in overweight adults [35]; or how the initial amino acid intake influences phosphorus and calcium homeostasis in preterm infants [36]. Hemp protein is known to have high content of arginine [13], and its value in plasma was actually increased (+25%), which could be one of the reasons why supplementation with this protein source could be helpful in adequately modulating lipid metabolism and overall health status. In fact, there is a favorable link between improved insulin sensitivity and zinc serum concentrations in obese patients affected by arginine [37]. For this reason, evaluating plasma-circulating amino acids following specific nutritional interventions is of interest in order to comprehend the mechanisms in physiological changes.

It was observed that, overall, the patients at the end of the intervention compared to the patients prior to it had higher levels of AAAA (+2587.6%), Pea (+99.2%), and Mhis_1 (+57.5%) and lower levels of asparagine (−47.7%) and alanine (−29.9%). These three amino acids, which highly increased after the intervention, were not correlated with the biochemical parameters (Figure 1). However, the levels of AAAA found in the intervention did not correlate with the results obtained by Wang et al. [38], who indicated that this compound is associated with diabetes risk and is a potential modulator of glucose homeostasis in humans. The plasma-circulating AAAA was extremely increased at the end of the intervention, but considering the individual values coming from the patients, the relevance of the data might not be reliable, as the values are close to zero. In addition, in this study, the correlation between glucose and AAAA was found to be 0.099 (Figure 1), indicating that they were not associated directly under the conditions assayed. Pea is a nonessential, phosphorous-bearing amino acid which can be found in foodstuff such as soybean, eggs, and milk [39], and can be also formed endogenously from serine via phosphorylation of ethanolamine. Low levels of phosphoethanolamine are generally associated with a need to ingest magnesium or a protein deficiency, and it can imply an affection of the parasympathetic nervous system because of the absence of the neurotransmitter acetylcholine [40]. The higher levels of Pea are likely to be associated with the extra protein intake derived from hemp, which might have been involved in an improvement of the health statuses of the patients, similarly to the reported increase in Mhis_1, which has been indicated as a marker of a high-protein diet [41].

The main amino acid that muscles quantitatively release is alanine, which the liver can convert to glucose more readily than any other amino acid [42]. A decrease in alanine in plasma levels might be of interest in terms of glucose modulation, although these levels were not found to be decreased. However, the relationship with the aerobic exercise must be also noted, considering that, for instance, Ueda et al. [43] reported that an amino acid mixture enriched with arginine, alanine, and phenylalanine stimulated fat metabolism during exercise. It has been described that the association between alanine and incident T2DM might be driven by an increased glutamate level [44]. It has been described that a high asparagine-to-aspartate ratio is associated with markedly increased risk of T2DM [45], but in the data obtained and after the processing of the data, the values of aspartate were not considered relevant. However, considering that the asparagine values highly decreased, the ratio of both was decreased, which might be linked, consequently, to a potential decrease in the risk of developing T2DM. Asparagine was correlated with the LDL-C values, which represent a risk factor for metabolic diseases. This was also observed by Luo et al. [45], indicating how a decrease in asparagine might go together with a decrease in LDL-C. It has been also reported that the bioavailability of asparagine affects glycolytic and thermogenic activities in adipose tissues, providing a potential nutritional strategy to enhance systemic energy homeostasis [46]. It is thought that variations in the rates of protein digestion are reflected in the plasma amino acid response. Based on the dynamics of the plasma amino acid reactions, the idea of “fast” and “slow” proteins was developed in response to variations in the rates of digestion [47].

Finally, nutrition; metabolic rate; and interactions between the metabolism of amino acids, carbohydrates, and lipids all affect the status of amino acids. Metabolic modulation might be unraveled by analysis of the plasma amino acids profile together with indicators of glucose and lipid metabolism [47]. The statistical analysis of the correlation between amino acids and minerals and biochemical parameters revealed some direct and inverse correlations, which might be of interest clinically, although considering the experimental design of this intervention, it is difficult to draw conclusions. According to the data, a high correlation between histidine and LDL-C was found (value > 0.51), and the correlation with the rest of the parameters, except for glucose, was also considerable, indicating that there might be a positive relationship between histidine in plasma and amelioration of metabolic parameters. In this sense, different authors have been describing how histamine (coming from histidine) signals and abnormal lipid metabolism are somehow related, although it is not clear how, and this requires further exploration [48]. Similarly, sarcosine has been described to serve as a crucial link between the metabolism of amino acids and lipids, and mechanistic investigations show that it is a powerful stimulant of basal macroautophagy [49]. In this study, the levels of sarcosine increased by around 20% and were moderately correlated with the biochemical parameters, corroborating this relationship. Increases in sarcosine have been related to delays of aging and correlated with dietary restrictions [49], as was the case in this study.

According to a meta-analysis evaluating supplementation with taurine in humans, this amino acid had no discernible impact on serum lipids, blood pressure, or body composition in diabetic patients. It was helpful in lowering glycemic indices such as HbA1c, fasting blood sugar, and HOMA-IR [50]. In this study, there was an increase in this amino acid, although not highly correlated with any of the parameters. The reduction in urea was consistent with the improvement in the biochemical parameters, as increased values of blood urea are related to renal damage. Insulin-secreting defects associated with chronic kidney disease arise from elevated circulating levels of urea that increase islet protein O-GlcNAcilation and alter glycolysis [51]. Information about threonine is not very consistent among different studies, and consequently, it is not easy to elucidate its role, if any, in glucose homeostasis or the process of delaying the development of diabetes [52], whereas D-serine causes diet-independent hyperglycemia due to reduced insulin secretion from pancreatic beta cells [53]. Ottosoon et al. [44] quantified 35 plasma metabolites (including amino acids) in 1049 individuals without coronary heart disease and diabetes, from which 204 developed T2DM after a follow-up period of 6.1 years. These authors found strong correlations of numerous amino acids with blood lipid values (HDL-C and TAG), as well as with fasting glucose levels and BMI. According to the authors, glutamate was the most strongly associated metabolite for developing T2DM, followed by increased levels of branched-chain amino acids (i.e., valine, leucine, and isoleucine) and decreased levels of asparagine. Similarly to this, it has been proposed elsewhere that branched-chain amino acids and aromatic amino acids (i.e., phenylalanine and tyrosine) could be potential biomarkers of the disruption of glycolipid metabolism [54]. In this study, glucose levels were decreased after the intervention, which could be linked to avoiding the development of T2DM similarly to the decrease in valine (−24.3%), a branched-chain amino acid, indicating that the subjects subjected to the nutritional intervention might have a lower risk of developing T2DM if they followed the diet and exercise proposed.

Samman et al. [47], studying the postprandial state response in a randomized cross-over design trial with 10 healthy volunteers ingesting protein, reported that some amino acids could be related to specific metabolic responses. Specifically, they were able to find correlations between plasma glucose concentrations and alanine, lysine, and histidine, whereas TAG was correlated with ornithine and tyrosine. Also, arginine was related to plasma zinc concentrations during the postprandial period, but this oligoelement was not considered in this study. The differences in correlations might be due to the health status of individuals, as being healthy or suffering from specific conditions would change the metabolic responses. Similarly, a report by Bagheri et al. [55] identified a metabolic pattern (including 19 metabolites) associated with obesity. From such a pattern, compounds such as alanine, glutamate, proline, tyrosine, and branched-chain amino acids were higher in obese participants, while asparagine and serine were higher in non-obese subjects. Along the same lines, Long et al. [56] identified differences in the amino acid contents in the serum and plasma of patients with diabetes and prediabetes. In the case of prediabetic adults compared to healthy individuals, higher levels of isoleucine, alanine, proline, glutamate, AAAA, and lysine were found, while lower levels were found for glycine, serine, and citrulline. In this study, decreases in alanine (−29.9%) and glutamate (−10.7%) were found, potentially linked to a reduced risk of developing pre-diabetes, since alanine was correlated with the values of TAG, total cholesterol, and LDL-C, as also described by Ueda et al. [35]. Circulating concentrations of phenylalanine and tyrosine are increased in adults suffering from metabolic syndrome (obese states that are insulin-resistant or have T2DM). In metabolomics studies, variation in plasma phenylalanine concentration (which was modestly increased in T2DM) has contributed to the separation of non-diabetics compared to participants with T2DM in PCA [57]. In this study, the phenylalanine concentration at the end of the intervention was decreased (−23.0%), which is in line with the separation described by Adams et al. [57] in patients and healthy adults, indicating through a cross-sectional study that these two amino acids were somehow related and affecting the insulin resistance condition. This is consistent with our results, where phenylalanine was correlated with the TAG and HDL-C content, which are relevant parameters related to the insulin condition [58].

Overall, the correlation of amino acids with the improvement of specific biochemical parameters or the minerals content is difficult to draw, as the nutritional intervention was not designed for that. However, for some correlations, corroborated information has been found in the literature, increasing the weight of evidence on how plasma-circulating amino acids might impact the health statuses of humans. In this study, the effectiveness of hemp protein supplementation within a context of Mediterranean diet together with regular aerobic exercise on the metabolic statuses of overweight individuals was assessed. The biological relevance of hemp protein in human studies is not widely reported. Recently, Samsamikor et al. [59] reported how the consumption of hemp seed protein for 6 weeks in 35 adults with mild hypertension could lower both angiotensin-converting enzyme and renin activities and raise the NO concentration in plasma compared with casein. However, in relation to blood lipids and circulating amino acids, little information has been reported, to the authors’ knowledge [14]. This study [14] investigated the effects of hemp protein consumption on glycemic response and satiety. Participants in the study consumed hemp protein (20 or 40 g), soy protein (20 or 50 g), or carbohydrates as controls. The results demonstrated that both hemp and soy proteins significantly lowered postprandial blood glucose levels compared to the carbohydrate control, with no significant difference between the effects of the two protein types. These findings indicate that hemp protein can positively influence the glycemic response, suggesting potential benefits for human well-being.

The amount of evidence in relation to the benefits of hemp protein consumption in humans is still limited, and the results obtained in this study indicate the potential of this source of protein to modulate human metabolism. However, a number of limitations need to be acknowledged in this study: the target population only encompassed 23 subjects, and no healthy subjects were included in the study. The reported mean values may have elevated standard deviations due to plasma amino acid levels that were nearing the lower limits of detection in certain cases. The meals contained relatively small amounts of protein, and if more protein had been ingested, it is probable that additional variations in the amino acid response would have been noticed.

## 5. Conclusions

In conclusion, the findings of this paper underscore the significant impact of plant protein supplementation as a crucial component of a healthy lifestyle regimen, particularly for overweight individuals. Under the conditions assayed, in the target population, it was found that a hypocaloric diet supplemented with hemp protein combined with moderate physical exercise decreased BMI and abdominal fat, while at the same time improving the lipid profile. Through comprehensive analysis of plasma-circulating amino acids and health status parameters, this study illuminates the positive outcomes associated with incorporating plant-based protein sources into dietary practices. By enhancing amino acid profiles and contributing to improved health markers, such as weight management and metabolic health, plant protein supplementation emerges as a promising strategy for promoting overall well-being in this population. It can be proposed that lifestyle changes could delay the development of metabolic syndrome, ameliorating the health status of the patients, potentially linked to the changes in the plasma concentrations of specific amino acids. These results not only advocate for the integration of plant proteins into dietary guidelines, but also emphasize the importance of personalized nutritional approaches tailored to individual needs. Moving forward, further exploration and implementation of such interventions hold substantial promise for mitigating the burden of overweight-related health complications and fostering sustainable lifestyle modifications that are conducive to optimal health outcomes. However, more studies with higher populations (and with patients with different metabolic impairments and healthy individuals), as well as the assessment of other parameters, are required in order to understand the underlying mechanisms behind the changes reported and the correlations among the parameters.

## Figures and Tables

**Table 1:** Anthropometric characteristics of the subjects before (t_0_) and after (t_f_) the intervention. Values are expressed as the arithmetic mean and confidence intervals (95% CI) of the subjects (n = 23, 35% males). *p* value < 0.05 implies a significant difference.

Variable	t_0_ (95% CI)	t_f_ (95% CI)	*p*-Value
Weight (kg)	91.19 (84.89–97.49)	82.20 (76.42–87.97)	<0.001
BMI (kg/m^2^)	31.88 (29.90–33.86)	28.73 (26.78–30.68)	<0.001
Abdominal circumference (cm)	103.23 (99.27–107.20)	91.87 (88.07–95.68)	<0.001
Body Fat (%)	39.08 (35.76–42.41)	35.77 (32.31–39.22)	<0.001
SBP (mmHg)	123.39 (118.44–128.34)	117.56 (112.05–123.07)	0.01718
DBP (mmHg)	81.17 (77.46–84.87)	76.82 (73.07–80.57)	0.006432
Body Water (%)	43.55 (41.32–45.77)	45.34 (43.08–47.59)	<0.001
Bone Mass (Kg)	2.77 (2.55–3.00)	2.65 (2.46–2.85)	0.001574
Basal Metabolism (kcal)	1697.0 (1556.98–1837.62)	1594.78 (1474.14–1715.42)	<0.001
Muscle Mass (%)	34.58 (29.37–39.78)	32.61 (28.26–36.97)	<0.001
Visceral Fat (%)	10.76 (9.18–12.33)	8.97 (7.44–10.51)	<0.001
Beats per minute	71.52 (66.93–76.11)	73.08 (67.95–78.22)	0.3346
Metabolic Age (years)	57.08 (53.14–61.02)	53.73 (48.86–58.61)	0.009444

BMI: body mass index; SBP and DBP: systolic and diastolic blood pressure.

**Table 2:** Blood biochemistry values of the subjects before (t_0_) and after (t_f_) the intervention. Values are expressed as the arithmetic mean and confidence intervals (95% CI) of the subjects (n = 23, 35% males). *p* value < 0.05 implies a significant difference.

Variable	t_0_ (95% CI)	t_f_ (95% CI)	*p*-Value
Glucose (mg/dL)	84.82 (77.09–92.54)	87.23 (83.82–90.63)	0.5411
TAG (mg/dL)	130.23 (94.40–166.06)	65.40 (54.24–76.57)	<0.001
Total Cholesterol (mg/dL)	207.26 (191.90–222.63)	146.89 (131.53–157.01)	<0.001
HDL-C (mg/dL)	47.48 (41.29–53.66)	72.29 (59.79–84.79)	<0.001
LDL-C (mg/dL)	133.73 (120.09–147.38)	61.51 (49.00–74.01)	<0.001

TAG: triacylglycerides; LDL-C and HDL-C: low and high-density lipoprotein cholesterol.

**Table 3:** Serum oligoelement values of the subjects before (t_0_) and after (t_f_) the intervention. Values are expressed as the arithmetic mean and confidence intervals (95% CI) of the subjects (n = 23, 35% males). *p* value < 0.05 implies a significant difference.

Variable	t_0_ (95% CI)	t_f_ (95% CI)	*p*-Value
Calcium (mg/dL)	13.75 (12.81–14.68)	11.79 (11.27–12.32)	<0.001
Phosphorus (mg/dL)	13.07 (11.45–14.69)	5.65 (5.38–5.92)	<0.001
Chloride (nMol/L)	96.39 (92.02–99.86)	104.67 (101.98–107.37)	<0.001
Magnesium (mg/dL)	1.97 (1.90–2.04)	2.01 (1.97–2.06)	0.3741

**Table 4:** Plasma circulating amino acid values of the subjects before (t_0_) and after (t_f_) the intervention. Values are expressed as the arithmetic mean and confidence intervals (95% CI) of the subjects (n = 23, 35% males). *p* value < 0.05 implies a significant difference.

Variable	t_0_ (95% CI) (μmol/L)	t_f_ (95% CI) (μmol/L)	*p*-Value
Phser	7.63 (7.08–8.18)	6.91 (6.11–7.70)	0.1324
Taur	14.88 (12.04–17.72)	16.87 (13.58–20.15)	0.3382
Pea	0.86 (0.62–1.11)	1.46 (1.01–1.91)	0.01695
Urea	1004.02 (926.99–1081.06)	808.57 (716.13–901.01)	0.001193
Thr	28.05 (25.54–30.55)	23.94 (21.37–26.51)	0.008221
Ser	24.62 (22.59–26.66)	22.82 (20.48–25.17)	0.1611
Asn	23.80 (21.56–26.05)	12.01 (10.78–13.25)	<0.001
Glu	10.21 (8.09–12.32)	8.21 (3.49–12.93)	0.004339
Gln	102.75 (95.52–109.98)	137.19 (118.20–156.17)	<0.001
AAAA	1.23 (0.82–1.63)	1.07 (0.76–1.39)	0.482
Gly	47.61 (41.86–53.37)	43.70 (36.67–50.73)	0.03543
Ala	85.12 (77.98–92.25)	57.51 (52.40–62.63)	<0.001
Aaba	4.81 (4.24–5.38)	4.83 (4.24–5.42)	0.9566
Val	49.43 (45.17–53.69)	36.54 (33.25–39.82)	<0.001
Cys	8.33 (7.51–9.15)	7.96 (7.20–8.72)	0.3419
Met_Cysth	5.45 (5.04–5.87)	4.08 (3.74–4.42)	<0.001
Ile	14.51 (13.06–15.97)	10.74 (9.68–11.79)	<0.001
Leu	28.86 (25.99–31.73)	27.80 (25.75–29.84)	0.3178
Tyr	16.97 (15.60–18.34)	13.56 (12.69–14.43)	<0.001
Phe	12.93 (12.05–13.80)	9.68 (9.10–10.26)	<0.001
Amm	39.53 (36.87–42.19)	28.27 (25.04–31.49)	<0.001
Orn	21.57 (19.14–24.00)	17.92 (16.13–19.70)	0.00242
Lys	38.83 (36.56–41.10)	39.34 (36.09–41.78)	0.7381
Mhis_1	3.18 (2.40–3.95)	3.72 (2.68–4.75)	0.56
His	17.04 (16.24–17.84)	13.24 (12.52–13.96)	<0.001
Trp	6.32 (5.46–7.18)	6.56 (5.51–7.61)	0.72
Arg	8.73 (6.91–10.55)	9.27 (8.11–10.43)	0.4954
Sarc	15.35 (14.40–16.29)	18.42 (16.87–19.97)	0.002875
Citr	5.58 (5.09–6.06)	4.46 (4.04–4.89)	<0.001

## Data Availability

Data described in the manuscript, code book, and analytic code will be made available upon request (e.g., application and approval, payment, other). Requests for data of the study can be sent to Prof. Sergio Montserrat-de la Paz, Department of Medical Biochemistry, Molecular Biology, and Immunology, School of Medicine, University of Seville, Spain (E-mail: delapaz@us.es).

## Supplementary Materials

The following supporting information can be downloaded at: https://www.mdpi.com/article/10.3390/nu16111594/s1, Table S1: Anthropometric characteristics of the subjects before (t_0_) and after (t_f_) the intervention.; Table S2: Blood biochemistry values of the subjects before (t_0_) and after (t_f_) the intervention; Table S3: Serum oligoelement values of the subjects before (t_0_) and after (t_f_) the intervention; Figure S1: Participant flow chart of the nutritional intervention; Figure S2: (A) PCA of the raw amino acid data and (B) PCA of the amino acid dataset after preprocessing and imputation of missing values by half the minimum value; Figure S3: Results of the application of the sPLS-DA technique to the amino acid dataset. (A) Representation of the first two components of the generated optimal model.

## Abbreviations

AAAA, α-aminoadipic acid; BMI, Body Mass Index; HDL, high-density lipoprotein; LDL, low-density lipoprotein; Mhis_1, 1-metylhistidine; PCA, Principal component analysis; Pea, Phosphoethanolamine; PLsDA, Sparse Partial Least Squares Discriminant Analysis; ROS, Reactive oxygen species; T2DM, Type II Diabetes Mellitus; TAG, triacylglycerides.
