# Supplementary material for: Mediterranean Diet Combined with Regular Aerobic Exercise and Hemp Protein Supplementation Modulates Plasma Circulating Amino Acids and Improves the Health Status of Overweight Individuals
Source: Nutrients. 2024 May 23;16(11):1594. doi: 10.3390/nu16111594 (PMC11174559; doi:10.3390/nu16111594)

Table S1. Anthropometric characteristics of the subjects before (t_0) and after (t_f) the intervention. Values are expressed as the arithmetic mean and confidence intervals (95% CI) of the male subjects (*n* = 8) and female subjects (*n* = 15). *P* value <0.05 implies a significant difference.

**Variable**		**t_0 (95% CI)**		**t_f (95% CI)**	***P*-value**
**Weight** (Kg)	Female	87.10 (80.75-93.44)	Female	79.29 (72.84-85.74)	<0.001
	Male	98.87 (84.39-113.35)	Male	87.65 (74.76-100.53)	
**BMI** (Kg/m²)	Female	32.36 (29.84-34.87)	Female	29.40 (26.84-31.97)	<0.001
	Male	30.98 (26.94-35.03)	Male	27.47 (23.90-31.04)	
**Abdominal circumference** (cm)	Female	100.53 (95.64-105.42)	Female	90.23 (85.15-95.30)	<0.001
	Male	108.31 (101.64-114.98)	Male	94.96 (88.54-101.38)	
**Body Fat** (%)	Female	43.21 (40.86-45.56)	Female	39.84 (36.66-43.02)	<0.001
	Male	31.35 (25.74-36.95)	Male	28.13 (23.37-32.90)	
**SBP** (mmHg)	Female	120.26 (115.62-124.90)	Female	113.80 (108.54-119.05)	0.017
	Male	129.25 (117.104-141.39)	Male	124.62 (111.61.137.63)	
**DBP** (mmHg)	Female	80.60 (77.04-84.15)	Female	77.73 (73.59-81.87)	0.006
	Male	82.25 (72.14-92.35)	Male	75.12 (65.97-84.27)	
**Body Water** (%)	Female	40.60 (39.12-42.07)	Female	42.62 (40.61-44.63)	<0.001
	Male	49.08 (45.79-52.37)	Male	50.43 (47.34-53.52)	
**Bone mass** (Kg)	Female	2.48 (2.37-2.58)	Female	2.40 (2.32-2.48)	0.002
	Male	3.33 (2.92-3.74)	Male	3.12 (2.73-3.51)	
**Basal Metabolism** (kcal)	Female	1522.06 (1449.84-1594.29)	Female	1451.53 (1390.03-1513.03)	<0.001
	Male	2025.87 (1751.51-2300.23)	Male	1863.37 (1607.72-2119.03)	
**Muscle mass** (%)	Female	27.77 (26.58-28.95)	Female	26.74 (25.79-27.68)	<0.001

	Male	47.35 (36.76-57.93)	Male	43.63 (35.26-52.00)	
**Visceral Fat** (%)	Female	9.80 (8.27-11.32)	Female	8.40 (6.70-10.09)	<0.001
	Male	12.56 (8.74-16.37)	Male	10.06 (6.43-13.68)	
**Beats per minute**	Female	73.66 (67.20-80.13)	Female	76.26 (69.88-82.65)	0.335
	Male	67.50 (61.40-73.59)	Male	67.12 (58.16-76.08)	
**Metabolic Age** (years)	Female	59.33 (54.56-64.10)	Female	55.66 (49.70-61.62)	0.009
	Male	52.87 (45.31-60.43)	Male	50.12 (40.04-60.20)	

Table S2. Blood biochemistry values of the subjects before (t_0) and after (t_f) the intervention. Values are expressed as the arithmetic mean and confidence intervals (95% CI) of the male subjects (*n* = 8) and female subjects (*n* = 15). *P* value <0.05 implies a significant difference.

**Variable**		**t_0 (95% CI)**		**t_f (95% CI)**	***P*-value**
**Glucose** (mg/dL)	Female	84.02 (73.09-94.96)	Female	86.56 (81.85-91.27)	0.541
	Male	86.31 (73.91-98.70)	Male	88.48 (82.71-94.24)	
**TAG** (mg/dL)	Female	98.77 (81.78- 115.76)	Female	61.52 (49.80-73.25)	<0.001
	Male	189.22 (91.76-286.69)	Male	72.67 (44.87-100.48)	
**Total Cholesterol** (mg/dL)	Female	205.17 (188.11-222.23)	Female	148.35 (138.26-158.44)	<0.001
	Male	211.19 (173.41- 248.97)	Male	144.14 (117.13-171.15)	
**HDL-C** (mg/dL)	Female	51.65 (43.25-60.05)	Female	83.05 (66.42-99.68)	<0.001
	Male	39.65 (32.44-46.85)	Male	52.12 (43.39-60.85)	
**LDL-C** (mg/dL)	Female	133.76 (116.6-150.92)	Female	52.99 (39.70-66.28)	<0.001
	Male	133.69 (104.60-162.78)	Male	77.48 (50.68-104.28)	

Table S3. Serum oligoelement values of the subjects before (t_0) and after (t_f) the intervention. Values are expressed as the arithmetic mean and confidence intervals (95% CI) of the male subjects (*n* = 8) and female subjects (*n* = 15). *P* value <0.05 implies a significant difference.

**Variable**		**t_0 (95% CI)**		**t_f (95% CI)**	***P*-value**
**Calcium** (mg/dL)	Female	13.67 (12.45-14.89)	Female	11.50 (10.85-12.16)	<0.001
	Male	13.88 (12.03 -15.73)	Male	12.34 (11.39-13.30)	
**Phosphorus** (mg/dL)	Female	13.09 (11.03- 15.15)	Female	5.77 (5.40-6.15)	<0.001
	Male	13.05 (9.66-16.44)	Male	5.42 (5.03 – 5.81)	
**Chloride** (nMol/L)	Female	95.79 (90.73-100.84)	Female	105.03 (101.53-108.53)	
	Male	97.52 (92.68-102.37)	Male	104.01 (98.61-109.42)	<0.001
**Magnesium** (mg/dL)	Female	1.96 (1.87-2.04)	Female	2.02 (1.96-2.09)	0.374
	Male	2.01 (1.86-2.16)	Male	2.00 (1.92-2.07)	

Figure S1. Participant flow chart of the nutritional intervention.

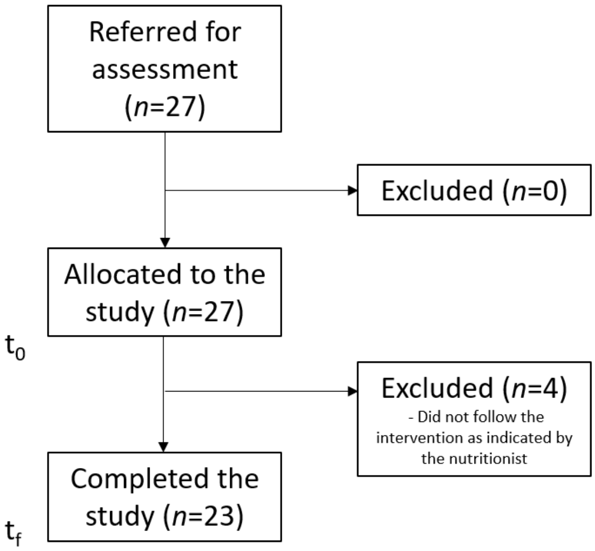

Figure S2 (A) PCA of the raw amino acid data and (B) PCA of the amino acid dataset after preprocessing and imputation of missing values by half the minimum value. The red colour corresponds to patient samples at the initial time of the intervention (t_0), and the blue colour corresponds to patient samples at the final time of the intervention (t_f).

(A)

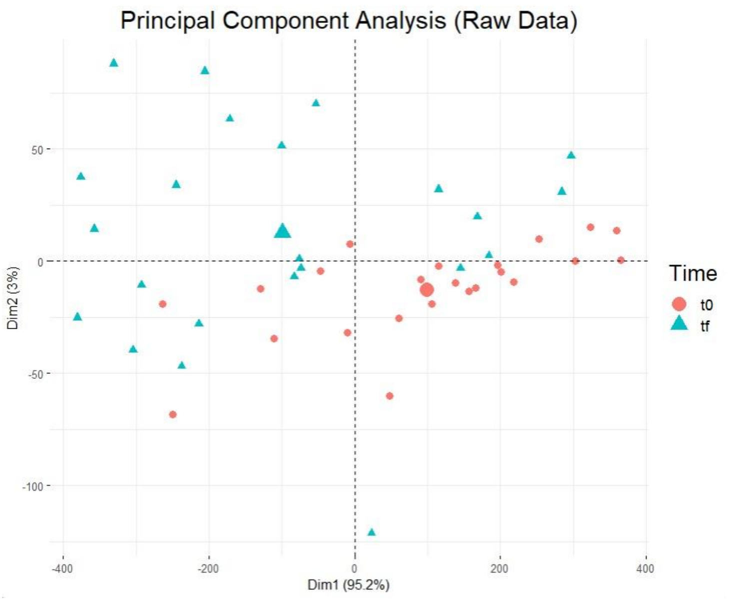

(B)

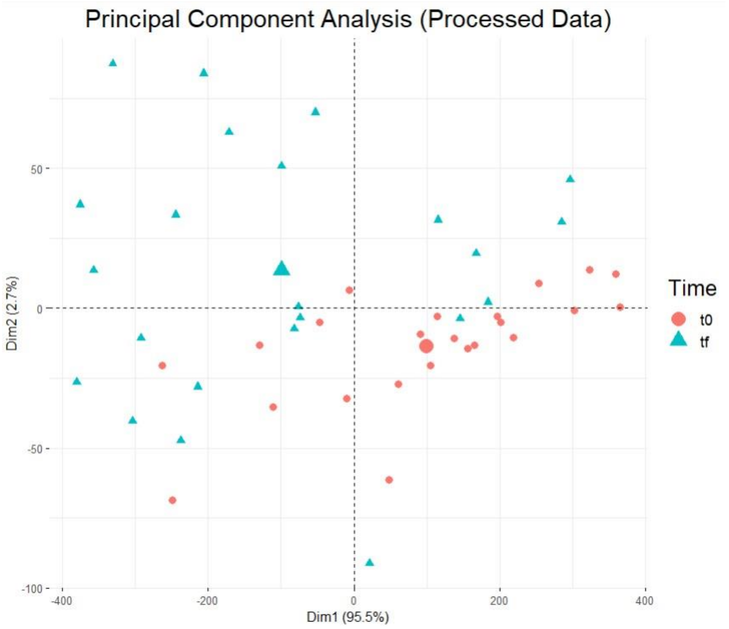

Figure S3. Results of the application of the sPLS-DA technique to the amino acid dataset. (A) Representation of the first two components of the generated optimal model. Component 1 (x-axis) explains 27% of the variance of the data, while component 2 (y-axis) explains 14% of the variance of the data. Component 1 is mainly responsible for the separation of both cohorts of patients, while component 2 is more involved in explaining the intra-group variance. (B) Values and directions of the loadings of the top ranked amino acids in component 1. The loadings represent the contribution of each amino acid to this component, indicating the importance of each one in the separation of the subjects at both times of the intervention. Phe, phenylalanine; Ala, alanine; His, histidine; Asn, asparagine.

(A)

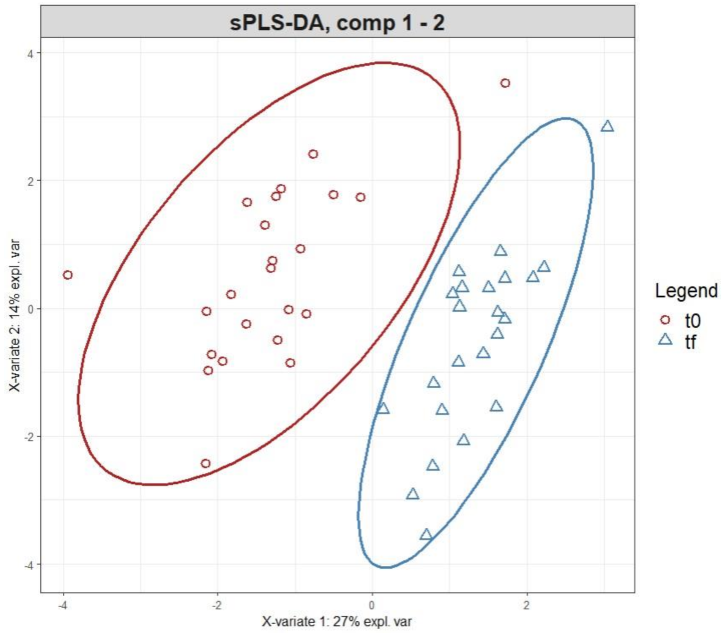

(B)

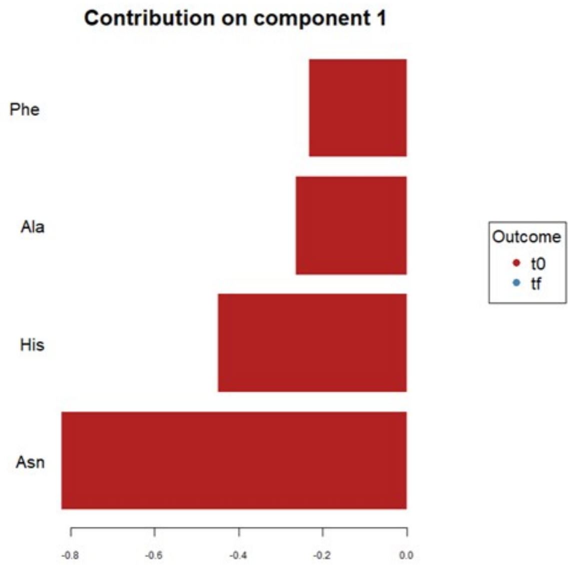

Supplement: Supplementary file 1 [file nutrients-16-01594-s001.zip › nutrients-3014485-supplementary.pdf]
